# In situ cryo-electron tomography reveals local cellular machineries for axon branch development

**DOI:** 10.1083/jcb.202106086

**Published:** 2022-03-09

**Authors:** Hana Nedozralova, Nirakar Basnet, Iosune Ibiricu, Satish Bodakuntla, Christian Biertümpfel, Naoko Mizuno

**Affiliations:** 1 Department of Structural Cell Biology, Max Planck Institute of Biochemistry, Martinsried, Germany; 2 Laboratory of Structural Cell Biology, National Heart, Lung, and Blood Institute, National Institutes of Health, Bethesda, MD; 3 National Institute of Arthritis and Musculoskeletal and Skin Diseases, National Institutes of Health, Bethesda, MD

## Abstract

Neurons are highly polarized cells forming an intricate network of dendrites and axons. They are shaped by the dynamic reorganization of cytoskeleton components and cellular organelles. Axon branching allows the formation of new paths and increases circuit complexity. However, our understanding of branch formation is sparse due to the lack of direct in-depth observations. Using in situ cellular cryo-electron tomography on primary mouse neurons, we directly visualized the remodeling of organelles and cytoskeleton structures at axon branches. Strikingly, branched areas functioned as hotspots concentrating organelles to support dynamic activities. Unaligned actin filaments assembled at the base of premature branches accompanied by filopodia-like protrusions. Microtubules and ER comigrated into preformed branches to support outgrowth together with accumulating compact, ∼500-nm mitochondria and locally clustered ribosomes. We obtained a roadmap of events supporting the hypothesis of local protein synthesis selectively taking place at axon branches, allowing them to serve as unique control hubs for axon development and downstream neural network formation.

## Teaser

Nedozralova et al. performed cryo-electron tomography on axon branches from mouse primary neurons and discovered concentrations of cellular activities at branching sites: ER spreading and ER comigration with microtubules, mitochondria fission, and protein synthesis in free and ER-attached polysomes.

## Introduction

The development of neurons with their extremely polarized structure and function is unique. Several long protrusions or neurites extend from the main cell body, the soma, where the nucleus is located. One of the protrusions develops into the axon, while the remaining protrusions develop into dendrites (Barnes and Polleux, 2009). Axons are functionally distinct from dendrites; dendrites receive signals from the axons of upstream cells via synaptic connections, whereas axons transmit signals to the dendrites of downstream cells. The molecular organization of the axon is uniquely suited to support local developmental processes, reflecting its specialized function. Within the backbone of the axon, microtubules (MTs) form parallel bundles with their plus ends oriented toward the distal end of the axon (Baas et al., 1988; Baas and Lin, 2011; Stepanova et al., 2003; van Beuningen and Hoogenraad, 2016). An actin-rich growth cone at the tip of a growing axon probes extracellular signaling molecules to identify the synaptic target on dendrites of an adjacent neuron (Dent and Gertler, 2003; Lowery and Van Vactor, 2009).

The long distance from the soma to the tip of an axon requires a regulatory system controlling local molecules (Dalla Costa et al., 2021; Goldberg, 2003; Holt et al., 2019; Stiess et al., 2010). This regulatory system includes differential expression of and enrichment for proteins that are critical for the local development and regulation of axonal homeostasis. In particular, clusters of mRNAs localized within dendrites and axons (Cioni et al., 2018; Holt et al., 2019; Poon et al., 2006; Taylor et al., 2009) indicate local protein synthesis. The types of locally enriched mRNAs depend on the developmental stage and their location within a neuron (Cioni et al., 2018). During the growth phase, mRNAs coding for proteins of the synthesis machinery such as ribosomes and for cytoskeletal components, which are needed to extend the cell, are found predominantly in growth cones (Bassell et al., 1998; Zivraj et al., 2010). This type of regulation has also been observed in distal axons during regeneration (Gumy et al., 2010). However, despite the critical role of local translation in axons, actions of the translation machinery are not well understood. Although there has been growing evidence for the presence of ribosomes along axons (Koenig et al., 2000; Noma et al., 2017; Tcherkezian et al., 2010), it has been a major challenge that there was no direct observation of protein synthesis accompanied by cytoskeleton and organelle reorganization. Direct observations of the local axonal environment at a molecular level aid in our understanding of neuronal growth and local reorganization.

During the development of the nervous system, axon branching serves to propagate signals to diverse regions of the nervous system (Kalil and Dent, 2014). Axon branching begins with the formation of actin-rich filopodia, short cellular protrusions, resulting from a signaling pathway that is induced by extracellular cues (Bodakuntla et al., 2021; Spillane et al., 2013; Tang and Kalil, 2005; Wang et al., 1999). Filopodia are the structural precursors of axon branches, and they develop into mature branches by the recruitment of MTs to the filopodia (Dent and Kalil, 2001; Gallo, 2011; Gallo, 2013). At the axon branch point, there is an enrichment of mRNAs (Spillane et al., 2013) encoding proteins such as β-actin (Wong et al., 2017) that may be required to form the initial premature branched axon (Donnelly et al., 2013). Interestingly, it has also been reported that mitochondria are enriched at axon branching points (Spillane et al., 2013), which may provide energy (Sheng, 2017) and may adjust the Ca^2+^ concentration for signal transduction and branch morphogenesis (Hutchins and Kalil, 2008). While the information for those individual components is available, the orchestration that controls the organized assembly of the protein synthesis machinery, organelles, and the cytoskeleton at axon branches is largely unknown.

To understand the organization of the key players for axon branching, we directly visualized the molecular organization of both premature and mature axon branching sites of mouse primary neurons by cryo-electron tomography (cryo-ET). We show the localization of small, ∼500-nm mitochondria and short actin fragments at the branches. An intricate network of ER membranes was often found between MT bundles and mitochondria, occasionally wrapping around the other components. ER was generally found comigrating with MTs to the mature axon branch, accompanying the maturation process of the axon branch. We further demonstrate the first direct observation of the accumulation of ribosomes at axon branches. In some cases, clustered ribosome attached to meshed-planar ER membranes as ER tubes widened, spreading into the space made for the branching activity. Subtomogram averaging and distance analysis indicated that the clustered ribosomes formed active polysomes. Isolated ribosomes, possibly monosomes synthesizing a small number of proteins (Su et al., 2016), were only sparsely seen. This is in stark contrast to ribosomes found at synapses, where the majority of ribosomes formed monosomes (Biever et al., 2020), highlighting the requirement for different types and amounts of newly synthesized proteins at various local environments within an axon. Our observations provide a comprehensive picture of the axon branching process.

## Results and discussion

### Structural analysis of branching axons

**Figure 1.**
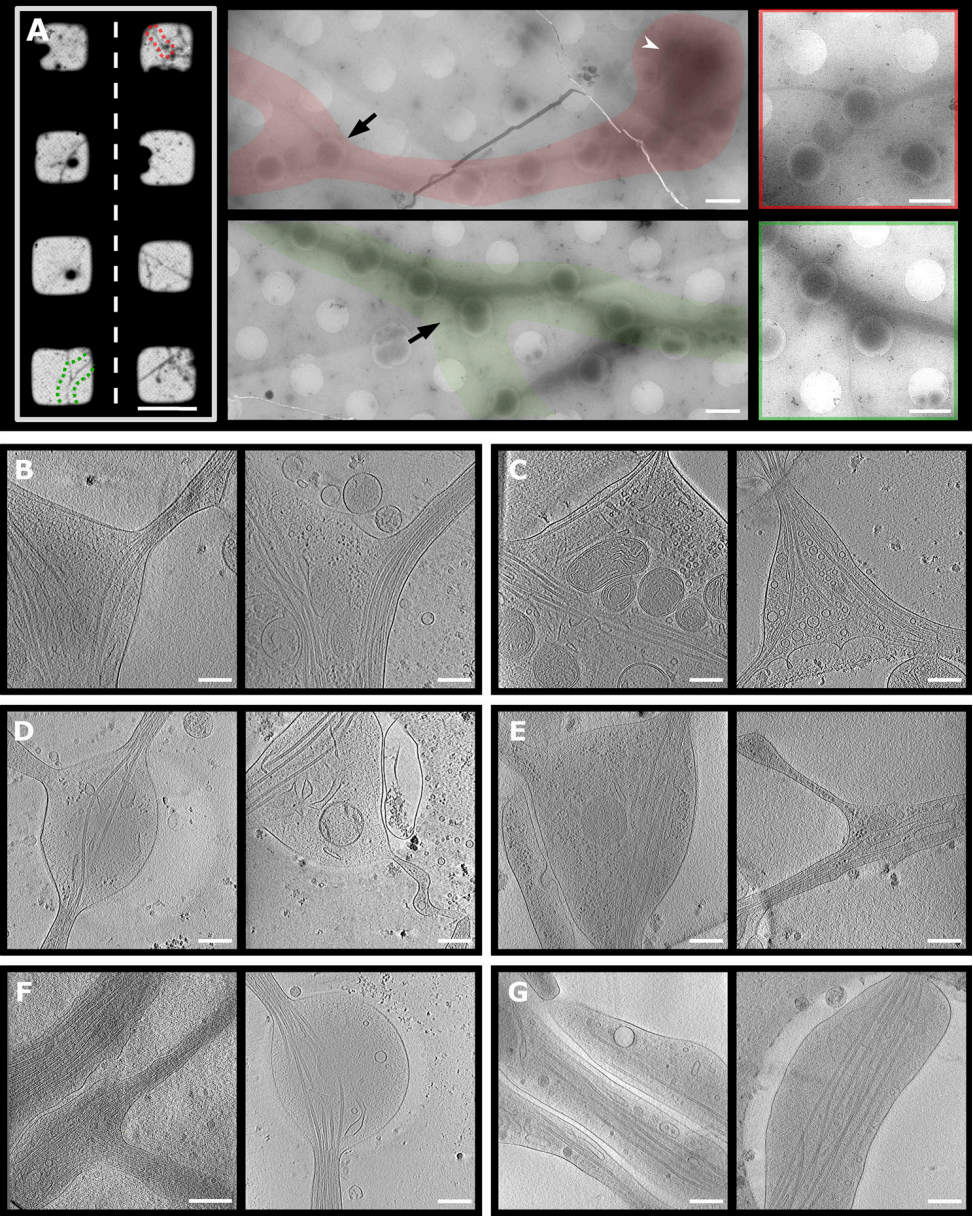
**Targeted areas for data collection of axon branches and representative snapshots of cryo-ET. (A)** Selection of areas for collection of tomograms. Left: Parts of cryo-EM map of grid with hippocampal neurons, selected area of interest in red and green. Middle: Low-magnification montages of areas depicted on grid map. Black arrow, branch region; white arrowhead, cell body. Right: Magnified area containing axon branch. **(B–G)** Slices from axon tomograms: mature branches from hippocampal (B) and thalamus (C) neurons; premature branches from hippocampal (D) and thalamus (E) neurons. Axon shafts from hippocampal (F) and thalamus (G) neurons. Scale bars: 100 µm (A left); 1.5 µm (A center and right); 250 nm (B–G).

**Figure S1.**
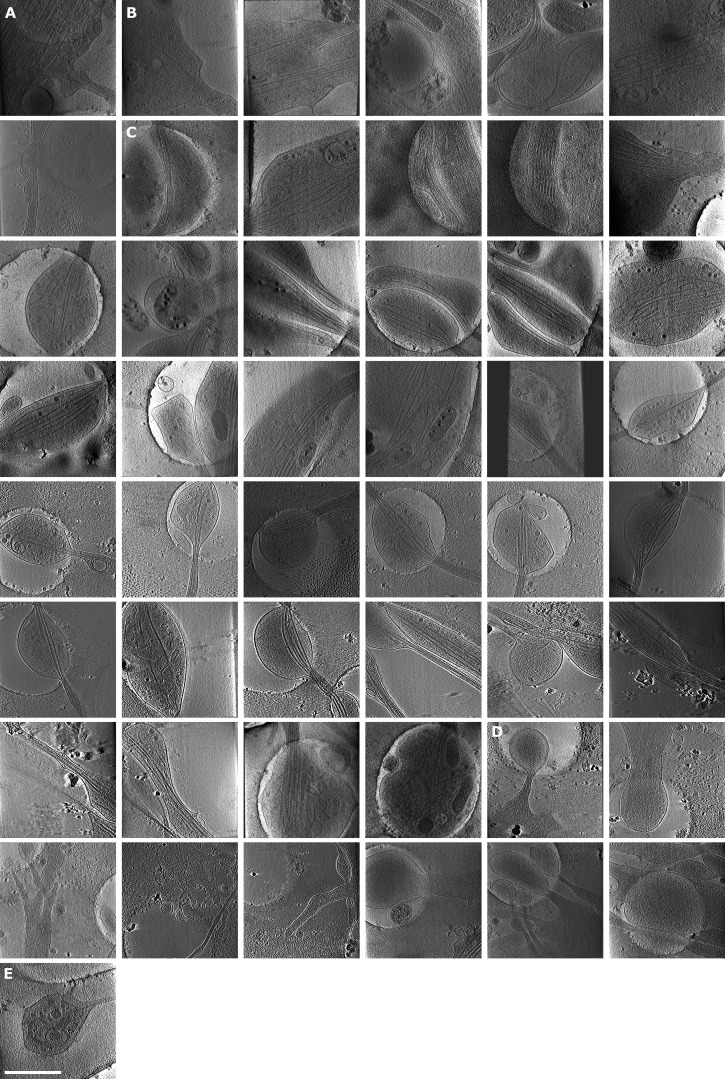
**Examples of mitochondria fission, ER entering branch, thin ER tubes, and ER-bound ribosomes. (A)** Mitochondria undergoing fission. White arrowheads depict ER pinching at mitochondria membrane or wrapping around the mitochondria. **(B)** ER tube present in axon branch together with MTs. Red arrowheads, ER; white arrowheads, MTs; black arrow, direction of new branch; white arrow, direction of main axon growth. **(C–G)** Analysis of ER diameter. In C, ER tube reaching extremely thin diameters; red arrowheads follow continuous ER tubes, and white arrowheads depict different ER. In D–F, ER (white arrowheads) wrapping around MTs (D), cross-section of ER tube (white arrowhead) wrapping around MTs (E), and diameter of thinnest ER tubes found in tomograms (median diameter 7.19 nm, *n* = 19; F). **(G)** Diameter of ER tube wrapping around MT (median diameter 12.21 nm,* n* = 29). **(H)** Slices of tomograms with ribosomes on the surface of ER. The area of ER is highlighted in yellow. Scale bars: 100 nm (A, B, H); 50 nm (C–E).

**Figure S2.**
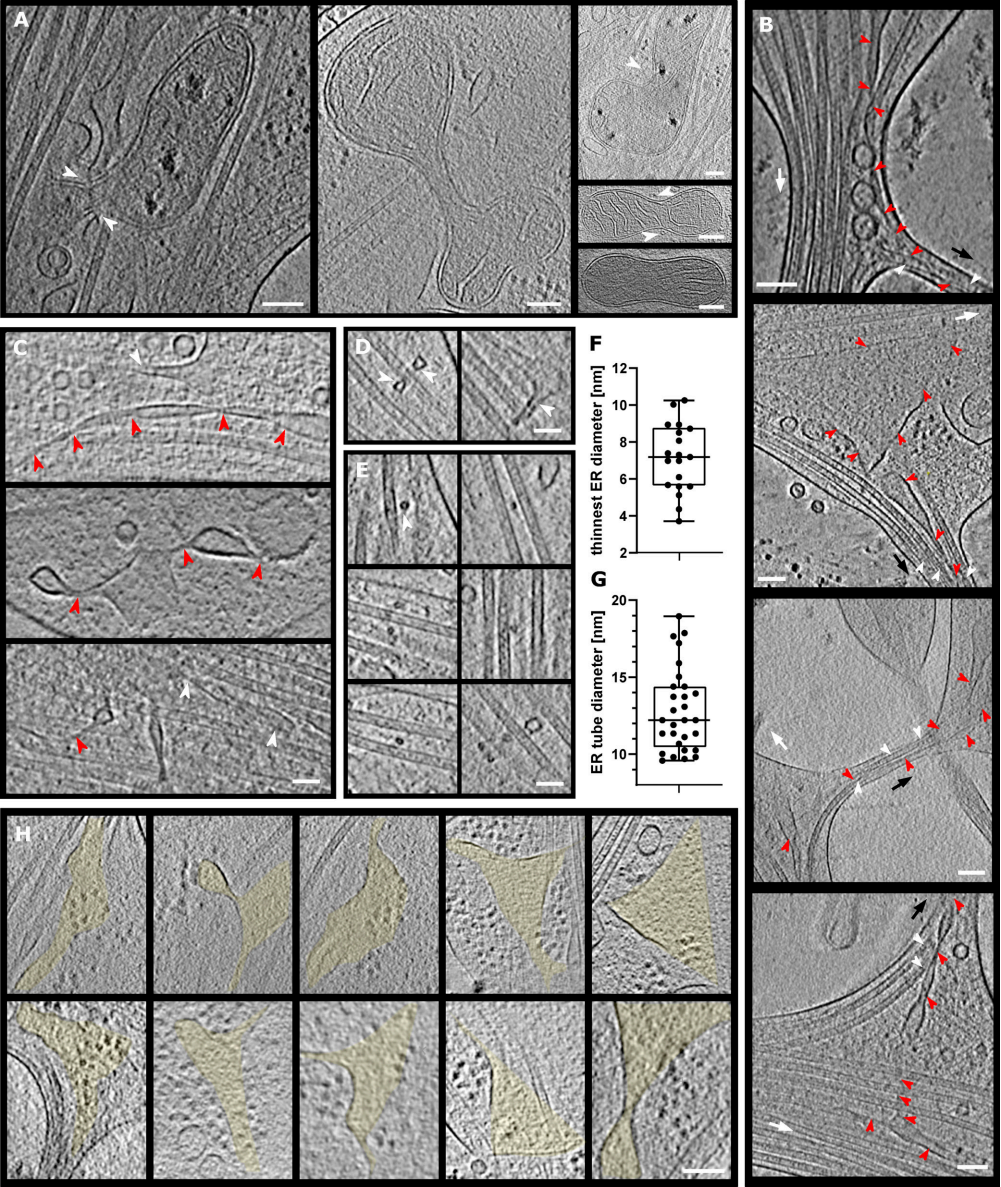
**Gallery of analyzed tomograms, part 1.** Snapshots of tomograms not shown in main figures. **(A–D)** Mature branches from hippocampal neurons (A), mature branches from thalamus neurons (B), premature branches from hippocampus (C), and premature branches from thalamus (D). Area of tomograms, 2 × 2 µm. Scale bars: 1 µm.

**Figure 2.**
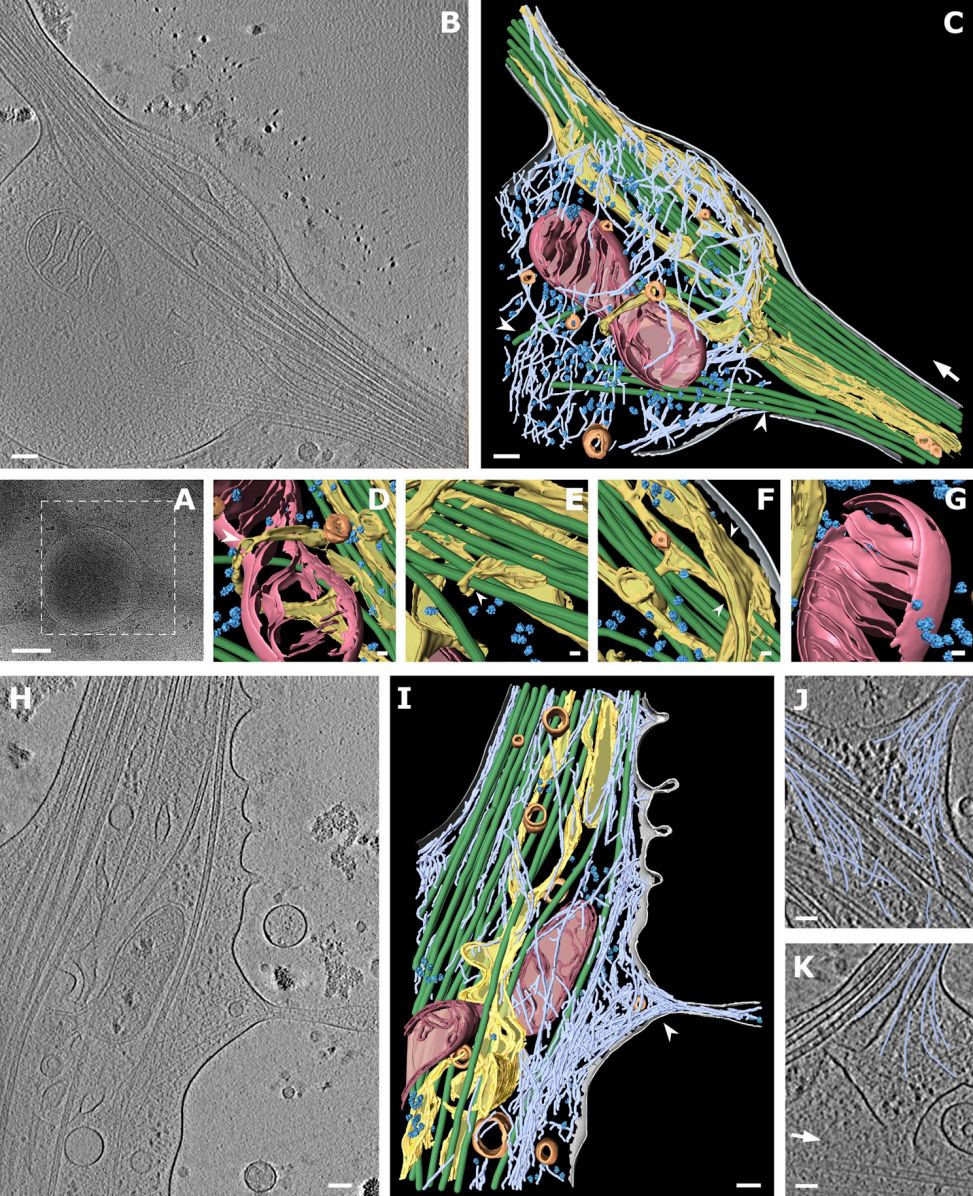
**Cryo-ET of mature and premature axon branches. (A)** Low-magnification view of a mature axon branching site. White box depicts area of tomographic data collection shown in B. **(B)** Slice of the tomographic reconstruction of the branching site. **(C)** Segmentation of the 430-nm thick tomographic volume from B. Color code: gray, cellular membrane; green, MTs; light blue, actin; pink, mitochondria; yellow, ER; dark blue, ribosomes; orange, vesicles. White arrow shows the direction of bundled MTs following axon growth; white arrowheads depict MTs entering the branch. **(D–G)** Zoomed-in views of segmented volume: ER wrapping around mitochondrion (white arrowhead; D), ER wrapping around MTs (E), ER forming a flat sheet (white arrowhead; F), ribosomes in the vicinity of mitochondrion (G). **(H)** Slice from tomographic reconstruction of a premature branch. **(I)** 353-nm segmented volume of tomogram (H; same color code as in C); white arrowhead depicts filopodium filled with actin. **(J)** Actin arrangement in the filopodia of a premature branch. **(K)** Actin arrangement in a mature branch (traced actin in light blue; white arrow shows direction of main axon growth). Scale bars: 500 nm (A); 100 nm (B, C, H, and I); 25 nm (D–G); 50 nm (J and K).

**Figure 3.**
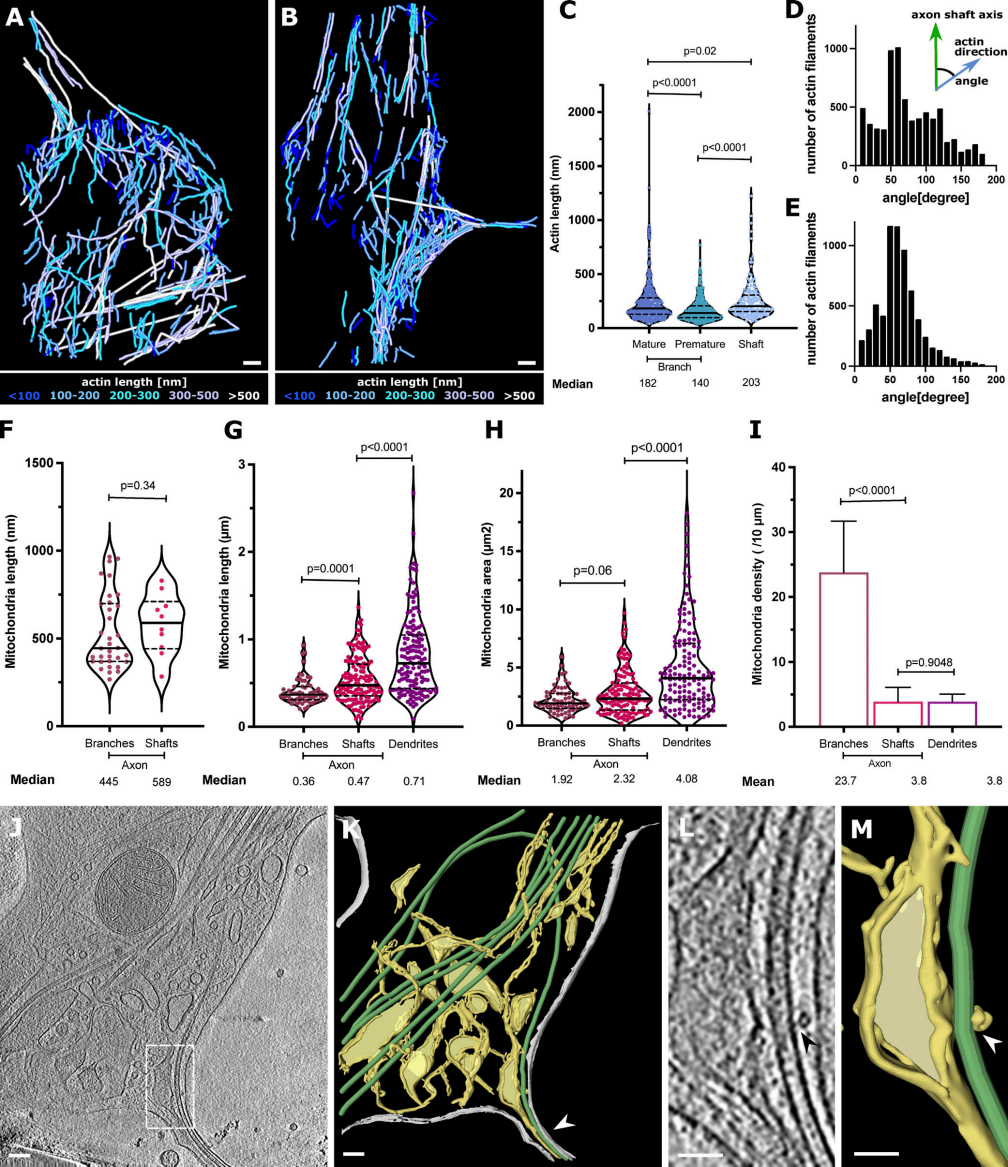
**Actin, mitochondria, and ER at axon branch. (A and B)** Analysis of actin present in mature (A) and premature (B) branch from Fig. 2, colored according to filament length. **(C)** Distribution of actin lengths at mature and premature branches and axon shafts, shown as scatter plots with interquartile range (*n* = 325 mature, *n* = 414 premature, *n* = 148 shaft). **(D and E)** Distribution of actin orientations from A (D) and from B (E). **(F)** Mitochondria length in tomograms (*n* = 34 branch, *n* = 10 shaft). Length is defined as the longest longitudinal axis of a single mitochondrion. **(G–I)** Mitochondria analysis from live-cell imaging of neurons showing mitochondrial length (G), mitochondrial area as box-and-whiskers graphs (H), and mitochondrial density as bar graphs (I) with mean ± SD (*n* = 63 branch, *n* = 119 shaft, *n* = 104 dendrite; significance was tested using Mann–Whitney *U* test). **(J)** Slice from tomogram showing various types of ER (thin tubes and flat sheets). **(K)** Segmented tomogram of J; color code: gray, cell membrane; green, MTs; yellow, ER; white arrowhead shows ER in branch together with MTs. White squares depict areas in L and M. **(L)** ER–MT contact (black arrowhead). **(M)** Segmentation of ER tube wrapping around MT (white arrowhead). Scale bars: 100 nm (A, B, J, and K); 50 nm (L and M).

To visualize the molecular organization of axons and axon branches, we prepared primary neuronal cell cultures from hippocampus and thalamus explants of mouse embryos at embryonic day 15.5 (E15.5). Various regions including axonal branches, axonal shafts, and growth cones were acquired at different magnifications to target areas for tomographic data collection (Fig. 1 A). We observed 119 tomographic reconstructions of axons (Table S1 and Figs. 1, S1, and S2). Among these reconstructions, 43 contained MTs in axon branch sites, indicating mature axon branches (Fig. 1, B and C; Fig. 2 B; and Video 1), and 23 nascent branches had membrane protrusions made of actin-containing filopodia but lacked MT reinforcements, indicating premature axon branches (Fig. 1, D and E; Fig. 2 H; and Video 2). For comparison, 53 tomograms were recorded in unbranched areas. At mature branches, MTs were tightly packed along axons (Fig. 2, B and C, arrow) similar as in axon shafts (Fig. 1, F and G), but were looser at branching points, where MTs spread apart to enter the branch. In mature branches, short unorganized actin filaments appeared to fill the space between MTs and plasma membrane (Fig. 2 C, light blue; Fig. 3, A, C, and D) with a median length of 182.4 nm (*n* = 325, Fig. 3 C), comparable to those found in axon shafts with a median length of 203.1 nm (*n* = 148, Fig. 3 C). In contrast, premature branches showed a dense, parallel actin network forming filopodia perpendicular to the axis of the axon, followed by the accumulation of short actin filaments with a median length of 140.3 nm (*n* = 414, Fig. 3 C) at the connection to the axon, while MTs ran along the axon (Fig. 2, H, J, and I, arrowhead; and Fig. 3, B, C, and E). Previous reports showed that actin forms predominant ring-like structures perpendicular to the axis of the axon (Vassilopoulos et al., 2019; Xu et al., 2013). However, due to the technical limitation of tomogram data acquisition (−60° to 60°), the information at the upper and lower areas is missing. This problem is known as “missing wedge” and likely constrains the visualization of actin rings.

**Video 1.** Tomogram of a mature branch. Slice view through tomographic volume with segmentation. Color code: gray, cellular membrane; green, MTs; light blue, actin; pink, mitochondria; yellow, ER; dark blue, ribosomes; orange, vesicles. Frame rate: 50 frames per second.

**Video 2.** Tomogram of a premature branch. Slice view through tomographic volume with segmentation. Color code: gray, cellular membrane; green, MTs; light blue, actin; pink, mitochondria; yellow, ER; dark blue, ribosomes; orange, vesicles. Frame rate: 50 frames per second.

### Organelle organization within axon branches

Besides the unique organization of the cytoskeleton, branching sites also showed a particular localization of mitochondria. Of the surveyed areas of 66 branches (43 mature and 23 premature) and 53 unbranched areas (43 axon shafts and 10 growth cones), we found 34 mitochondria among 22 mature and premature branches and 10 along axon shafts, indicating a strong enrichment of mitochondria in axon branches (Table S1). This tendency was also validated by light microscopy of mitochondria within whole neurons (Fig. 3, F–I; and Video 3).

**Video 3.** Mitochondria movements in axons and dendrites. Mitochondria were labeled with MitoTracker Red CMXRos (shown in red) and recorded at a time interval of 10 s. The outline of the cell was visualized by differential interference contrast every 10 s. **(A)** Representative video showing mitochondria movements in the axon shafts and branches. Mitochondria showed a tendency to accumulate at branches compared with axon shafts. Inset video highlights the mitochondria dynamic nature at the axon branches. **(B)** Representative video showing the mitochondria movements in cell body and dendrites. Mitochondria in the dendrites are longer in comparison to axonal mitochondria.

Our analysis showed that mitochondria in axon branches had a median length of 445 nm (n = 34), while those in axon shafts were measured to be 589 nm (n = 10; Fig. 3 F). Whole-neuron measurements using light microscopy (Fig. 3, G–I; and Video 3) showed that mitochondria along axons had a median length of 360 nm (*n* = 63) at branches and 470 nm (*n* = 119) in shafts, overall agreeing with cryo-EM observations. In contrast, mitochondria found in dendrites showed longer entities (710 nm, *n* = 104; Fig. 3 G), in agreement with previous studies showing that axonal mitochondria are significantly smaller than dendritic (Delgado et al., 2019; Lewis et al., 2018; Li et al., 2004; Popov et al., 2005). Mitochondria size may be related to the local Ca^2+^ concentration or to presynaptic neurotransmitter release (Lewis et al., 2018), which eventually leads to terminal axon branch formation. Branching axons contained several clustered mitochondria with a length of ∼500 nm (Fig. 1 C, left [EM]; Fig. 3 I [shown as density]; and Video 3 [live cell imaging]) and occasionally, we observed mitochondria undergoing fission (Fig. 2, C, D, H, and I; and Fig. S1 A). In our observations, mitochondria fission events happened at five axon branches while we did not observe that along axon shafts, suggesting that axon branching sites may act as preferred platforms for the duplication of mitochondria. Mitochondria were often found next to the ER network, and tubular ER was occasionally wrapped around mitochondria (Fig. 2, C, D, and H–I; and Fig. S1 A), either loosely (Fig. 2, C and D) or more tightly, contacting the wide surfaces of their membranes (Fig. 2, H and I). The wrapping by ER likely represents a stage of ER-facilitated fission of mitochondria (Lewis et al., 2016; Wu et al., 2017). However, we also observed mitochondria that underwent fission without attached ER, suggesting that ER wrapping may not be essential for mitochondria fission (Fig. S1 A). ER at branching sites took a planar mesh–like, spread-out form (Fig. 3, J and K), while it adopted a thin, tightly-packed tubular structure along axon shafts. The size of ER ranged from 4-nm thin tubes (Fig. S1, C and F) to wide flat areas >200 nm in width (Fig. 2 F, Fig. 3, J–M, and Fig. 5 H). ER membranes often intertwined with MTs (Fig. 2, E and F; and Fig. S1 D) or occasionally wrapped around the walls of MTs (Fig. 3, K and M; and Fig. S1 E). We found no density crosslinking MTs and ER membranes, presumably due to the low signal-to-noise ratio, though ER–MT connections can be made by molecules such as p180, CLIMP63, and kinectin (Cui-Wang et al., 2012; Farias et al., 2019; Shibata et al., 2010). Interestingly, we found that ER propagation to axon branches (Fig. S1 B) occurred for 41 mature branches of 43, but only for 3 of 23 premature branches, indicating that ER and MTs comigrated into the branching axons. ER membranes may promote the maturation and elongation of axon branches by serving as a lipid source for the growing plasma membrane. Comigrating ER and MTs may cooperate to grow stably toward branching axons, as they do in regular axon outgrowth (Farias et al., 2019).

### Ribosomes are in action at axon branches but not along stable axon shafts

**Figure 4.**
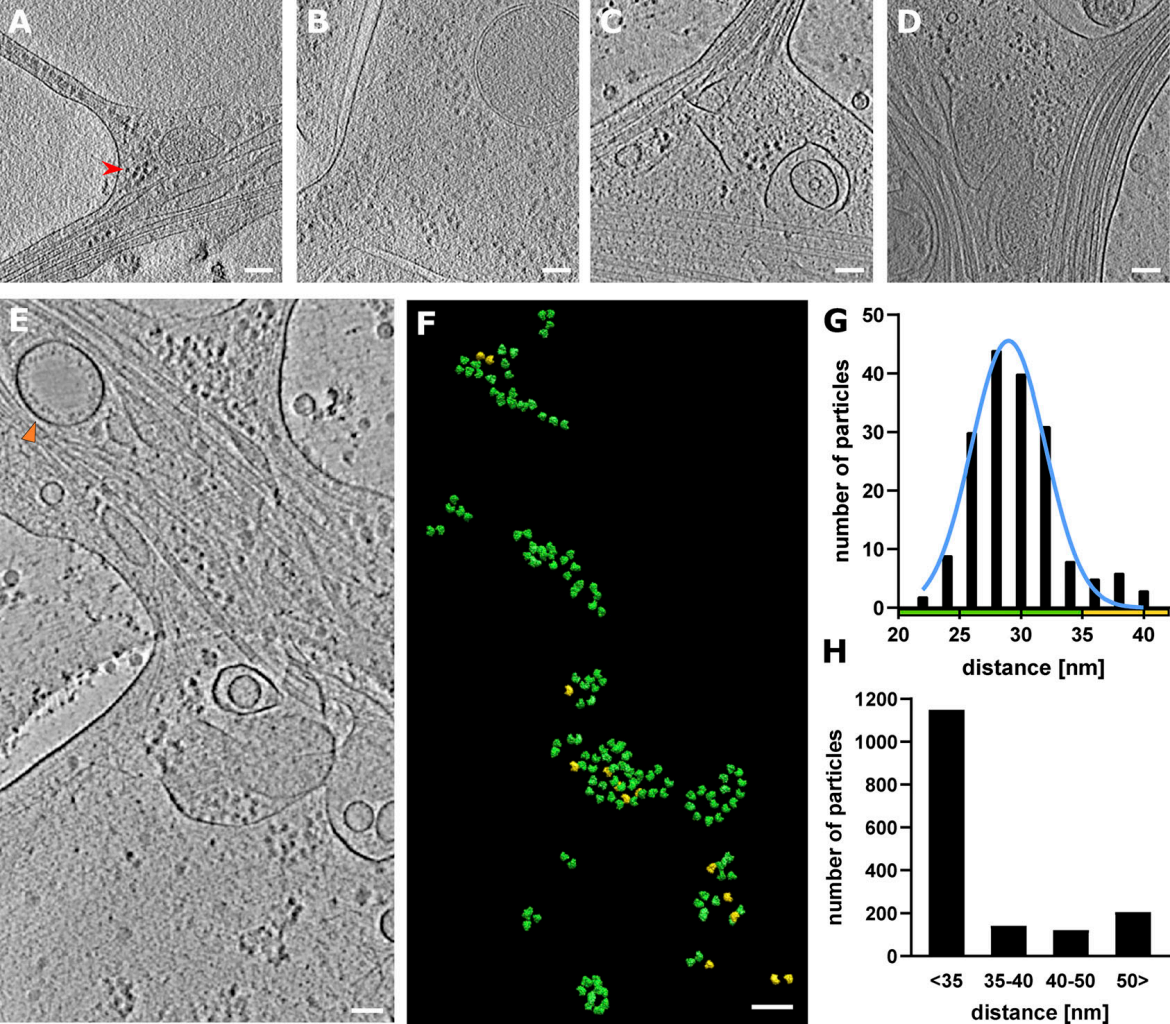
**Ribosome clusters at axon branch. (A and B)** Ribosomes at premature branch. Red arrowhead depicts an example of ribosome density. **(C and D)** Ribosomes at mature branch. **(E–G)** Analysis of distance between ribosomes. **(E)** Slice of analyzed tomogram (orange triangle shows vesicle with inner membrane densities). **(F)** Distribution of ribosomes in the 3D volume of tomogram in E, color code by distance between ribosome particle coordinates: green <35 nm, yellow >35 nm. **(G)** Distance distribution of ribosome particles in tomogram E. The graph shows particles with closest neighbor distance value <40 nm (*n* = 178). **(H)** Cumulative distance distribution for all analyzed ribosomes (*n* = 1,614).

**Figure S3.**
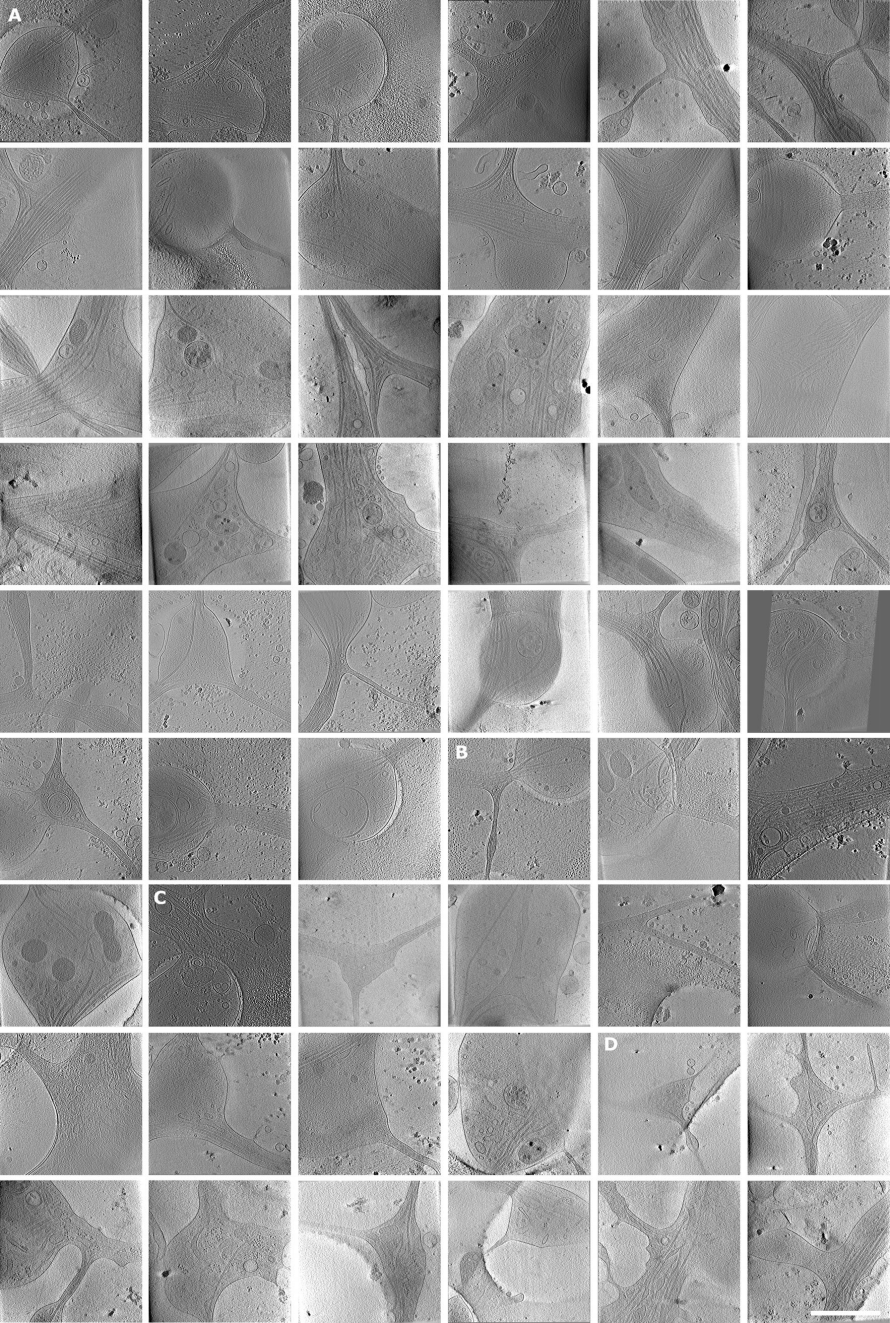
**Gallery of analyzed tomograms, part 2. (A–E)** Snapshots of tomograms. Premature branches from thalamus neurons (A), hippocampal neuron shafts (B), shafts from thalamus (C), tips of growth cones from hippocampus (D), and growth cone, tip of thalamus (E). Area of tomograms, 2 × 2 µm. Scale bars: 1 µm.

It is critical that neurons control their local environment due to their polarized and compartmentalized morphologies, with long distances between the soma and the tips of axons. To react rapidly to local requirements, specific areas of neurons may locally translate proteins (Holt et al., 2019); however, direct observation of localized protein synthesis has been challenging. Our tomographic observations of axons provide direct evidence of ribosomes in action (Figs. 1, 2, and 4), particularly, at axon branch sites, where we found accumulations of ribosomes in 30 of 66 axon branches. Ribosomes were found in 61% of premature and 37% of mature axons, indicating that ribosomes localized to developing axon branches. Ribosomes were also found in filopodia at the growth cone (Fig. S3, D and E). In contrast, we rarely observed ribosomes along the tightly packed axon shafts. The accumulated, closely packed ribosomes in axon branches were close to each other, with an average distance of 29.5 ± 3.4 nm (Fig. 4, F and G), agreeing with the reported distance between adjacent units in a polysome of 25–35 nm (Brandt et al., 2010; Brandt et al., 2009; Mahamid et al., 2016). These ribosomes likely represent a polysome arrangement, in which ribosomes are assembled along one strand of mRNA, sequentially synthesizing identical protein chains. The short distances between ribosomes were observed consistently in axon branches, with 70% of ribosomes falling within the 25–35 nm distance range (Fig. 4, G and H).

**Figure 5.**
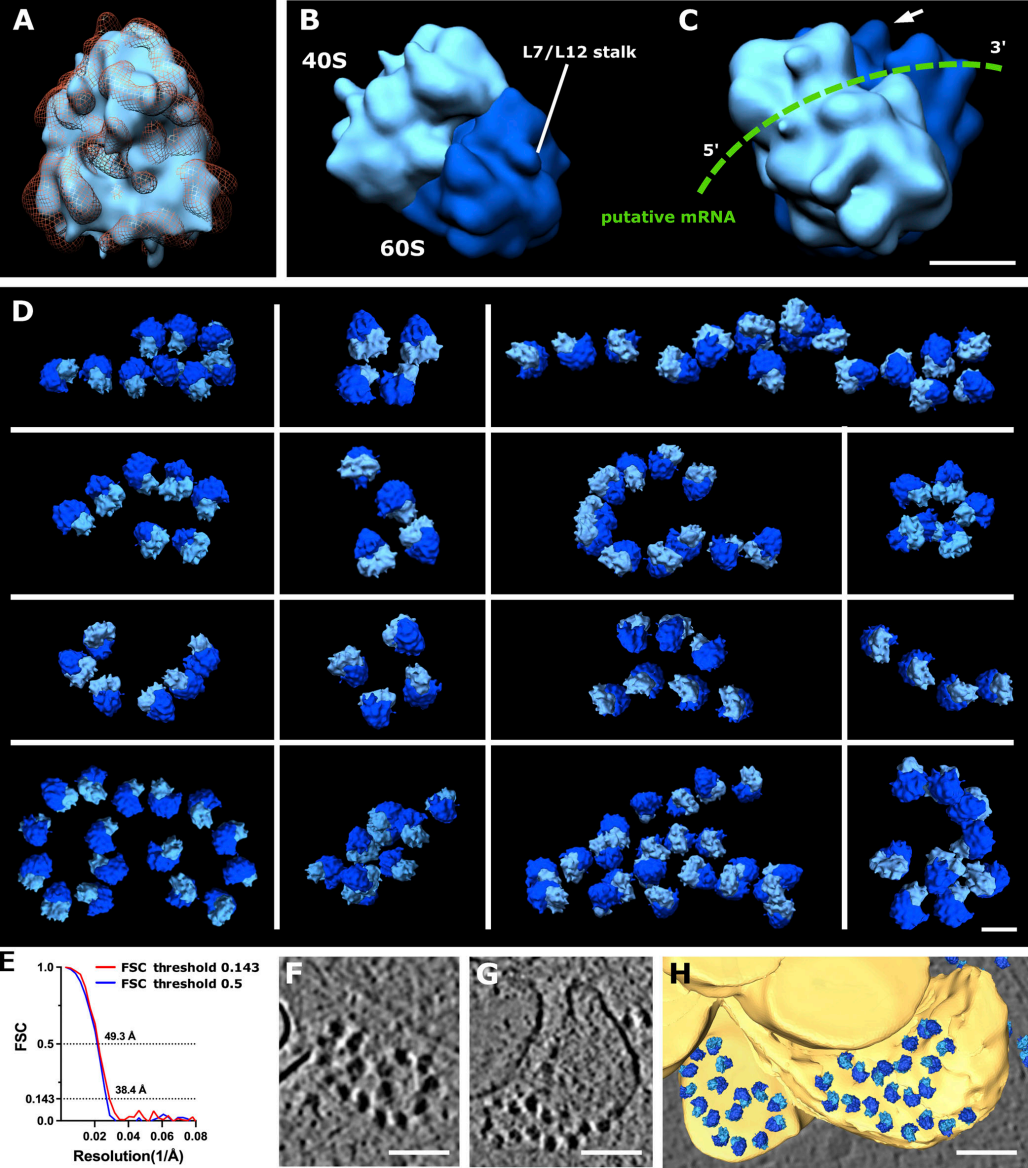
**Ribosome reconstruction and polysome orientation. (A)** Reconstructed ribosome (light blue) fitted into the 80S ribosome volume from EMD-3420 (orange mesh). **(B)** Reconstructed ribosome volume with depicted 40S (light blue) and 60S (dark blue) subunits and L7/L12 stalk. **(C)** Rotated view of ribosome from B. Putative path of mRNA depicted in green; white arrow shows L7/L12 stalk. **(D)** Polysomes found in various tomograms. **(E)** Estimation of reconstructed ribosome resolution, FSC curve. **(F and G)** Slices from tomogram of ribosomes on the surface of ER. **(H)** Segmented view of F and G. Scale bars: 10 nm (A–C); 25 nm (D); 100 nm (F–H).

To understand the molecular topology of ribosomes in axon branches, we computationally extracted 1,614 ribosomes and performed subtomogram averaging to a resolution of 38.4 Å with Fourier shell correlation (FSC) at 0.143 or 49.3 Å with FSC at 0.5 criteria (Fig. 5, A–C and E). The parameters of the orientation of individual ribosomes derived from the analysis were plotted back to the original tomograms to visualize their arrangements. Fig 5 D shows several representative ribosome arrays found in axon branches forming spiral-like shapes and resembling reported polysome arrangements (Brandt et al., 2010; Mahamid et al., 2016; Myasnikov et al., 2014). This analysis showed that the majority of ribosomes in branching axons was engaged actively in translation, synthesizing the same types of proteins.

Although most of the observed ER in axons had no ribosomes bound regardless of their morphology, we found ribosomes attached to the surface of planar areas of the ER mesh network (110 of 431 ribosomes analyzed in branching sites presented in Fig. 2; Fig. 5, F–H; and Fig. S1 H). The ER-bound ribosomes adopted a polysome-like organization, with a spiral shape similar to those in the cytoplasm. Those ribosomes may synthesize transmembrane proteins, as suggested by cell biological analysis, but they have not been observed by ultrastructural analyses yet (Merianda et al., 2009). When ER formed a thin tubular shape, there were no ribosomes attached to the membrane surface, presumably due to the high curvature. This also explains the absence of ribosomes along the axon shaft. Notably, 13% of ribosomes in branches were >50 nm from the nearest ribosome, too far to be considered a polysome. Although our observation does not offer to exclude that these ribosomes were in a resting state, not engaged in protein synthesis, it agrees with the notion that isolated ribosomes (monosomes) may be active in neurons, especially in synthesizing proteins that are only needed locally at low levels (Heyer and Moore, 2016; Holt et al., 2019). In comparison, the majority of ribosomes found at synapses are reported to form monosomes (Biever et al., 2020). The visualization of ribosomes provided direct evidence that proteins are synthesized locally in distal areas of axons, the site of dynamic cellular activities.

### Molecular orchestration at the axon branch

Neuronal polarization is a unique cellular developmental process that is of critical importance. The direct observation of this process is central to gain an overview of the orchestration of the involved players; however, it has been long missing. Here, using cryo-ET of mouse primary hippocampal neurons and thalamus explants, we obtained a direct close-up view of axon branches that have a remarkable local concentration of cellular machineries (cytoskeleton, ER, mitochondria, and ribosomes), which are critical for axon development and outgrowth. Axons are thin structures, filled with MT bundles; therefore, the space for cellular events is limited. That could be attributed to the fact that a mature axon is stable, with a tightly packed robust cytoskeleton. Axon branch points form cellular hubs, at which MT bundles bifurcate, providing space for additional cellular components and dynamic cellular events. In addition, the bifurcated cytoskeleton at branch points likely provides static reinforcement to hold cellular machineries in place. We found actual evidence of ongoing cellular activities, including active mitochondria fission, active ribosomes, spreading of ER, and short actin filaments. Future studies will address the mechanisms by which cellular machineries are recruited to and regulated at axon branches. The process of axon branching is critical for neural network formation during the development of the nervous system. Moreover, it plays a crucial role in neural homeostasis throughout the life cycle of the brain, including axon pruning during brain maturation and axon regeneration after brain injury. Elucidating the mechanisms governing the formation of axon branches will not only provide insights into fundamental neuronal processes but also build the basis for the understanding of neuronal circuit formation and function.

### Cytoskeleton remodeling and ribosome involvement

The stabilization of MTs within axons requires neuronal MT-associated proteins (MAPs) such as Tau, MAP7, and DCX (Baas et al., 2016; Chen et al., 1992; Moores et al., 2004; Tymanskyj and Ma, 2019), but at axon branching points, dynamic remodeling of MTs facilitates new branch formation (Yu et al., 2008). Among the factors involved in MTs remodeling at axon branches, we have previously reported a novel MT nucleation factor, SSNA1, which localizes at branch sites and promotes axon branching (Basnet et al., 2018). In vitro, we observed a surprising MT nucleation that facilitates the creation of lattice-sharing MT branches. Axon branching correlates with the MT-branching activity, leaving open questions about the remodeling of MTs during axon branching. While these questions were interesting to address, assessing MT bundles by ET was challenging because the branching axon was too thick to allow visualization of individual MTs within bundles. Determining how branched cytoskeleton bundles are made will be an important future area of research on axonal development.

The mechanisms that drive the rearrangement of cytoskeleton elements, MTs, and actin filaments inside axons and at axon branches are poorly understood. Actins are found sparsely along the axon shaft outside of MT bundles. At axon branching points, short, unaligned actins accumulated, accompanied by aligned actin filaments forming filopodia-based membrane protrusions. Where do these actins originate from? While it is possible that shorter actins are the depolymerization products of existing actin filaments at the shaft, actins are indicated to be locally synthesized during axon branch development (Spillane et al., 2013; Wong et al., 2017). Our data on the colocalization of short actins and active polysomes supports the hypothesis that local actin synthesis is a crucial part of the machinery to build up filopodia, which is a central process for cell shape formation. It would be revealing to find evidence of actin generation at local axon branching sites, for example, by direct observation of actin folding through its chaperone (Llorca et al., 1999). For the case of actin, we speculate that the local synthesis hub at the axon branching point would support dynamic cellular activities within the confined space of axon branches. In contrast, ribosomes were rarely found along the axon shaft, indicating that protein synthesis is tightly regulated within limited regions of the axon. How these ribosomes are regulated and accumulate at branching sites, distant from the soma, is unknown. Recently, it was reported that ribosomal protein components and their coding mRNAs are essential for axon branching (Shigeoka et al., 2019), providing a possible scenario that ribosomes themselves may be remodeled locally as well. Identifying the steps in ribosome remodeling in situ at branching points would be challenging. However, it would provide visual cues that could explain site-specific regulation of the molecular machinery that controls neuronal dynamics and the processes that lead to the establishment of new structures such as axon branches.

## Materials and methods

### Primary neuron cultures on EM grids

Quantifoil (R1/4 Au200, MultiA Au300, and R1.2/1.3 Au300) gold EM grids were plasma cleaned for 40 s and sterilized by UV light for 30 min. Grids were then coated with 1 mg/ml poly-L-lysine in 0.1 M borate buffer (Sigma-Aldrich) overnight, then washed three times in PBS and coated with laminin (Sigma-Aldrich) for 4 h (5 µg/ml for suspension culture and 20 µg/ml for explants). Grids were washed three times with PBS, covered with neurobasal/B27 medium, and incubated at 37°C.

Primary embryonic mouse neurons were prepared as either dissociated hippocampal cultures or tissue explants from thalamus. Neurons were prepared from E15.5 mice. Dissected hippocampi were placed into cold HBSS (HBSS supplemented with 1× Hepes, 1× Glutamax, and 1× penicillin and streptomycin) medium treated with trypsin and incubate at 37°C for 16 min, followed by washing with HBSS with FBS and then neurobasal/B27 medium followed by trituration. Thalamus tissue was placed into neurobasal/B27 medium, and explants were prepared by cutting thalamus into small pieces, which were incubated at 37°C for 30 min. 11 of the observed cryo-EM images of hippocampus neurons were from the effort of transducing MT-binding protein SSNA1. The transduction rate was low, and MT organization was not assessed in this study. The other components did not show any notable differences to the extent of our experimental evaluation. The dissociated cells were plated on coated EM grids at a concentration of 150,000 cells/ml and incubated at 37°C in 5% CO_2_. Explants were placed onto EM grids covered in neurobasal/B27 medium: NeuroBasal (Life Technologies) supplemented with 1× Glutamax, 1× B27 serum, and 1× penicillin and streptomycin. Half the culture medium was changed at 1 day in vitro (DIV1). Cell cultures were grown for 6–10 d. Then, EM grids with neuron cultures were manually vitrified in liquid ethane using a homemade plunger or vitrobot (Thermo Fisher Scientific).

### Cryo-ET data collection and processing

Cryo-ET data were collected on Titan Krios (Thermo Fisher Scientific) with a Gatan Quantum 967 LS and K2 Summit direct detector, an option of phase-plate, and an acceleration voltage of 300 kV. 66 tilt-series were collected using the phase-plate option with 0 defocus, and 53 tilt-series were collected without phase-plate at a defocus range of −3.5 to −5 µm. Tilt series were collected at −60° to 60° with a 2° angular increment in a dose-symmetric scheme using Serial-EM software (Hagen et al., 2017). The total electron dose was ∼90 e^−^/Å^2^, and the nominal magnification was 26,0000×, corresponding to a final pixel size of 5.46 Å. Images were taken in superresolution mode as 10-frame videos, and the video frames were aligned, combined, and dose-filtered using in-house frame alignment software implementing MotionCor2 (Zheng et al., 2017). A total of 119 tilt-series were assessed for this study, including 54 tilt-series of thalamus explants and 65 tilt-series of hippocampus neurons.

Individual images of the collected tilt-series were assessed manually, and low-quality images at the high tilt angle were removed from the dataset. Tilt-series were filtered according to the cumulative radiation dose (Grant and Grigorieff, 2015) and aligned on the basis of the patch tracking algorithm using the IMOD ETOMO package (Kremer et al., 1996). Tomograms were reconstructed from aligned stacks using weighted back-projection in IMOD (Mastronarde and Held, 2017). Tomograms were further 4× binned, resulting in pixel sizes of 21.8 Å. Tomograms were denoised by edge-enhancing diffusion (band command in Bsoft; Heymann et al., 2008).

### Tomogram segmentation

Tomograms were manually segmented using Amira software (Thermo Fisher Scientific). When applicable, membranes were segmented automatically using deconvolution filtering (Tegunov and Cramer, 2019) and a tool for membrane segmentation, TomoSegMemTV (Martinez-Sanchez et al., 2014). MTs were segmented manually in IMOD. Measurements of mitochondria length (44 mitochondria) and ER tube diameter (19 for thinnest ER measurements and 29 for ER tube diameter) were measured manually in IMOD. The obtained data are represented using box-and-whiskers graphs, in which each dot represents the measurement of an individual mitochondrion or ER, and the horizontal line indicates the median of the distribution. Data were plotted using Prism software (GraphPad).

### Actin analysis

For actin analysis, we analyzed 148 actins from axon shafts, 325 actins from mature branches, and 414 actins from premature branches. Actin was segmented manually using IMOD software. The IMOD model file generated was then converted into coordinate files. Each actin filament was then resampled into 10-nm spaced segments for further analysis. For each actin filament, two parameters were calculated: (a) angle of all 10-nm segments in the given actin filament and (b) length of each actin filament. For determination of angle, a vector pointing toward the longitudinal axis of a neuron was taken as a reference vector, and the angle between this vector and each actin segment was calculated using the Python3 NumPy library. Similarly, the length of each actin filament was calculated by adding the distance of all segments present in each filament using Python NumPy and SciPy libraries. The data was shown as scatter plots with median and interquartile range. Mann–Whitney *U* test was used to test the significance.

### Subtomogram averaging of ribosomes and distance analysis

1,614 ribosome particles from 11 unbinned tomograms that were collected with phase plate were picked using IMOD 3dmod software. The picked coordinates were then transferred into the subtomogram averaging package DYNAMO (Castaño-Díez et al., 2012), and ribosome averages were calculated as follows. The initial template used for the alignment was low-pass filtered to >100 Å (EMDB-5224). At that resolution, only the general shape and size of the ribosome was visible. The initial alignment was done using standard global settings from Dynamo, and the search space and angular increments were then gradually decreased for subsequent refinement. During refinement, subtomograms were split into odd and even half-sets, and Dynamo’s adaptive bandpass filtering was performed to avoid overfitting and estimate the attained resolution. The attained resolution was estimated by comparing the FSC of separately computed averages from odd and even half-sets. A bandpass filter was then applied in the next iteration based on this estimation. The final resolution was estimated to be 38.4 Å with the FSC 0.143 criterion and 49.3 Å with the FSC 0.5 criterion. We note that some of the high-tilt components may contain positive defocus, as the dataset was taken with 0 defocus option. However, with Volta phase plate, there is no contrast reversal with overfocus at low frequency, unlike the conventional approach (Fan et al., 2017). We also note that the resolution of ribosomes could be limited by the phase oscillation of the higher-frequency components. Distances between ribosome particles were calculated from refined coordinates from subtomogram averaging runs using Python3 (numpy and scipy libraries). For each particle, the closest neighboring distance was plotted into the distance distribution histogram and fitted with a nonlinear Gaussian curve in Prism. The orientation of ribosomes was assessed by placing reconstructed ribosome volumes into tomograms in CHIMERA using coordinates and alignment parameters derived from subtomogram averaging.

### Mitochondria imaging and analysis

Primary hippocampal neurons were cultured on glass-bottom dishes for 4 d in vitro at 37°C in 5% CO_2_. Mitochondria movements in the axons were performed in DIV4 neurons (Bodakuntla et al., 2020). DIV4 neurons were incubated with 2 nM MitoTracker Red CMXRos (#M7512; Thermo Fisher Scientific) for 1 min in the cell culture incubator and gently washed once with neurobasal conditioned medium (medium from untreated neurons). 1 ml of conditioned medium was added to the dish, and mitochondria movements in the axon were recorded using a Zeiss 880 confocal microscope with AiryScan superresolution module and the imaging chamber set to 37°C and 5% CO_2_. The microscope was controlled using Zeiss software. The videos were recorded with a 60× oil objective at a time interval of 10 s. The longest distance of individual mitochondria was defined as length, and it was measured using the ImageJ 2.3.0/1.53f particle analyzer plugin. Briefly, the first frame of the video was converted into binary and segmented using watershed algorithm, and then the particles were analyzed. The total numbers of mitochondria analyzed in different regions was 63 in axon branches, 119 in axon shafts, and 104 in dendrites. Mann–Whitney *U* test was used to test the significance of differences for mitochondria sizes and density.

### Statistical analysis

All numerical data corresponding to the cryo-ET are provided in Table S1. For the analysis of actin length and mitochondria size, the data are shown as dot scatter plots with median and interquartile range. In these plots, each dot represents the corresponding measurement value. The number of measurements for each graph is given in the corresponding figure legend. For analysis of mitochondria density in different regions of the neuron, the data are shown as bar graphs with mean ± SD. Significance of the data was tested using nonparametric Mann–Whitney *U* test. P values obtained for the comparisons were given directly on the graphs.

### Online supplemental material

Table S1 includes a numerical summary of analyzed tomograms. Fig. S1 contains examples of mitochondria fission, ER entering branch, thin ER tubes, and ER-bound ribosomes. Fig. S2 and Fig. S3 show a gallery of all analyzed tomograms that could not be included in the main text. Video 1 and Video 2 show representative tomograms of mature and premature axon branch, respectively. Video 3 includes light microscopy imaging of mitochondria movements in axons and dendrites.

## Supplementary Material

Table S1 is the numerical summary of analyzed tomograms. Analyzed parameters were sorted according to the brain region from which the neurons originated.

## Data Availability

Tomograms of premature and mature axon branches used in the figures were deposited to the Electron Microscopy Data Bank (EMDB) with accession codes EMD-25485 (premature axon branch) and EMD-25486 (mature axon branch). Additional tomograms used for the statistical analysis were deposited to the Electron Microscopy Public Image Archive (EMPIAR) with accession codes EMPIAR-10922 (mouse thalamus neurons) and EMPIAR-10923 (mouse hippocampus neurons).
