# Supplementary material for: In situ cryo-electron tomography reveals local cellular machineries for axon branch development
Source: J Cell Biol. 2022 Mar 9;221(4):e202106086. doi: 10.1083/jcb.202106086 (PMC8916118; doi:10.1083/jcb.202106086)
Supplement: Table S1 — is the numerical summary of analyzed tomograms. Analyzed parameters were sorted according to the brain region from which the neurons originated. [file JCB_202106086_TableS1.docx]

| **Tomograms according brain region** | | | |
| --- | --- | --- | --- |
| *Parameter* | *Thalamus* | *Hippocampus* | *Together* |
| **Branch** | **18** | **48** | **66** |
| Branch with mitochondria | 5 (28%) | 17 (35%) | 22 (33%) |
| Mitochondria fission in branch | 1 | 4 | 5 |
| Branch with ribosomes | 8 (44%) | 22 (46%) | 30 (45%) |
| Branch with mitochondria and ribosomes | 2 (11%) | 11 (23%) | 13 (19%) |
| **Axon shaft** | **35** | **8** | **43** |
| Shaft with mitochondria | 6 (17%) | 3 (38%) | 9 (21%) |
| Mitochondria fission in shaft | 0 | 0 | 0 |
| Shaft with ribosomes | 1 (3%) | 2 (25%) | 3 (7%) |
| **Growth cone** | **1** | **9** | **10** |
| Growth cone with mitochondria | 0 | 0 | 0 |
| Growth cone with ribosomes | 1 (100%) | 9 (100%) | 10 (100%) |
| **Total number of tomograms** | **54** | **65** | **119** |
| **Branch tomograms according to brain region** | | | |
| *Parameter* | *Thalamus* | *Hippocampus* | *Together* |
| **Mature branch** | **6** | **37** | **43** |
| Mature branch with mitochondria | 3 (50%) | 15 (40%) | 18 (42%) |
| Mitochondria fission in mature branch | 1 | 3 | 4 |
| Mature branch with ribosomes | 0 | 16 (43%) | 16 (37%) |
| Branch with mitochondria and ribosomes | 0 | 9 (24%) | 9 (21%) |
| ER inside mature branch | 6 (100%) | 35 (95%) | 41 (95%) |
| **Premature branch** | **12** | **11** | **23** |
| Premature branch with mitochondria | 2 (16%) | 2 (18%) | 4 (17%) |
| Mitochondria fission in premature branch | 0 | 1 | 1 |
| Premature branch with ribosomes | 8 (66%) | 6 (54%) | 14 (61%) |
| Branch with mitochondria and ribosomes | 2 (16%) | 2 (18%) | 4 (17%) |
| ER inside premature branch | 2 (16%) | 1 (8%) | 3 (13%) |
